# Phytochemical components analysis and hypolipidemic effect on hyperlipidemia mice of the aerial parts from *Allium sativum*

**DOI:** 10.3389/fnut.2024.1422857

**Published:** 2024-07-25

**Authors:** Bin Hu, Haibo Hu, Donghui Peng, Zheng Wei, Qiuhong Wang, Haixue Kuang

**Affiliations:** ^1^National Engineering Research Center for Modernization of Traditional Chinese Medicine-Hakka Medical Resources Branch, School of Pharmacy, Gannan Medical University, Ganzhou, China; ^2^Key Laboratory of Chinese Materia Medica, School of Pharmacy, Heilongjiang University of Chinese Medicine, Harbin, China; ^3^Ganzhou People's Hospital, Ganzhou, China; ^4^Guangdong Engineering Technology Research Center for Standardized Processing of Chinese Materia Medica, Guangdong Pharmaceutical University, Guangzhou, China

**Keywords:** *Allium sativum*, hyperlipidemia, mice, hypolipidemic, phytochemical components

## Abstract

**Background:**

The bulbs of *Allium sativum* are widely used as food or seasoning (garlic), while they have also been utilized as a famous traditional medicine since ancient eras for the treatment of scabies, tuberculosis, pertussis, diarrhea and dysentery, etc. However, very few studies focus on their abundant aerial parts, which are normally discarded during the harvest season.

**Methods:**

The hyperlipidemic mice model has been used to study the lipid-lowering effect of the aerial parts in this article. 180 mice were randomly divided into 18 groups, including blank control (BC), model (Mod), positive control (PC), and low-, medium-, and high-dose groups of the crude extract, petroleum ether, ethyl acetate, n-butanol, and residual water extracts (corresponding to CE, PEE, EAE, NBE, WE), with 10 mice in each group. The preventive effects of the extracts on hyperlipidemic mice lasted for four weeks. Ultra performance liquid chromatography quadrupole time-of-flight mass spectrometry (UPLC-Q/TOF-MS) and gas chromatography tandem mass spectrometry (GC-MS/MS) were used to analyze the chemical components of NBE and PEE respectively.

**Results:**

The results of the mice experiment showed that n-butanol extract (NBE) and petroleum ether extract (PEE) from the aerial parts could significantly reduce the contents of total cholesterol (TC), triglycerides (TG), low density lipoprotein cholesterol (LDL-C), alanine transaminase (ALT) and aspartate transaminase (AST) in serum of hyperlipidemic mice, and increase the contents of high density lipoprotein cholesterol (HDL-C). They could enhance the activity of superoxide dismutase (SOD) in liver and reduce the level of malondialdehyde (MDA). At the same time, they could improve steatosis and inflammation of liver cells. The results of phytochemical components analysis showed that NBE was rich in organic acids, flavonoids and nitrogen-containing constituents, while PEE contained organic sulfur compounds, aliphatic acids and derivatives, alkaloids, phytosterols, etc.

**Conclusion:**

These results support that the aerial parts of *A. sativum* are an interesting source of bioactive ingredients that may be useful in the prevention and treatment of hyperlipidemia.

## 1 Introduction

Hyperlipidemia is a chronic disease caused by abnormal lipid metabolism (1). It is characterized by the increase of triglyceride (TG), total cholesterol (TC), low density lipoprotein cholesterol (LDL-C) and/or the decrease of high density lipoprotein cholesterol (HDL-C) in the blood (2–4), and can cause a series of cardiovascular diseases such as atherosclerosis, stroke, coronary heart disease, myocardial infarction, hypertension, diabetes renal failure and Alzheimer’s disease (5–8). With the improvement of people’s living standards and changes in lifestyle, the incidence rate of hyperlipidemia is increasing year by year. How to safely and effectively prevent and treat hyperlipidemia, thereby preventing and treating cardiovascular diseases, has always been a hot research topic in the academic community. At present, the main lipid lowering drugs used clinically are statins, fibrates, etc. Although the effect are significant, recent studies have found that statins may cause adverse reactions such as liver injury, rhabdomyolysis, nephrotoxicity and diabetes (9, 10). Compared with chemical drugs, food-medicines are receiving increasing attention due to their low adverse reactions and high safety. The ancient Greek physician Hippocrates emphasized the important philosophical and medical foundation of diet in maintaining health and preventing and treating diseases (11). Both Eastern and Western ethnic groups have a tradition of using herbs in their diet to maintain health (12). Therefore, the use of food-medicines to prevent and treat chronic diseases such as hyperlipidemia has certain clinical value.

*Allium sativum* is a perennial herb of Liliaceae, more recently attributed to the family Amaryllidaceae (13). It is the oldest authenticated and most important food-medicine plant that has been used from ancient times as traditional medicine (14). The most commonly utilized parts of *A. sativum* are their bulbs (garlic), which have been used for cooking purposes as a spice that can flavor foods during the cooking process. Meanwhile, since ancient times, garlic has had a wide range of healthcare and therapeutic effects in clinical practice. Ancient medical literatures from China, Greece, Egypt, Rome and India each recorded medical applications for garlic (15). Scholars in the field of food and medicine have done a lot of research on garlic and more than 130 compounds have been isolated since the 1970s, mainly including organic sulfur compounds (14, 16–18), flavonoids (19), steroidal saponins (20), pyranone derivatives (21), cerebrosides (22), phenols (19), volatile oil (23, 24), polysaccharides (25, 26), amino acids, vitamins, and protein (14). The pharmacological activities of garlic are mostly focused on areas such as the treatment of cardiovascular disease (27, 28), the resistance to pathogenic microorganisms (29, 30), anti-tumor (31) and immune regulatory effects (32). Since the 1980s, a quantity of studies have been conducted on the regulating effect of garlic on blood lipids (33), and clinical trials have also confirmed its good lipid-lowering effect (34, 35).

Currently, extensive research has been conducted on the bulbs of *A. sativum*. However, there are few reports on the aerial parts of *A. sativum*, and only our group has conducted research on its active ingredients for anti platelet aggregation (36, 37). *A. sativum*, as an indispensable food-medicine plant, has a huge market demand and is cultivated in various countries around the world. However, only the bulbs are used, and thousand tons of the aerial parts are discarded after harvest, resulting in a great waste of resources. In order to fully develop and utilize the plant, it is necessary to conduct systematic research on the aerial parts.

In this paper, the hyperlipidemia mice model was established. By observing the body weight of the mice, calculating the liver index, detecting the contents of TC, TG, LDL-C, HDL-C, alanine transaminase (ALT) and aspartate transaminase (AST) in serum, measuring the level of malondialdehyde (MDA) and the activity of superoxide dismutase (SOD) in liver tissue, simultaneously conducting pathological examinations of the liver to explore the lipid-lowering effect of the aerial parts on hyperlipidemic mice. Furthermore, ultra performance liquid chromatography quadrupole time-of-flight mass spectrometry (UPLC-Q/TOF-MS) and gas chromatography tandem mass spectrometry (GC-MS/MS) were used to analyze the extraction components of *n*-butanol and petroleum ether with strong lipid-lowering activity, respectively.

## 2 Materials and methods

### 2.1 The aerial parts of *Allium sativum* samples and preparation

The aerial parts of *A. sativum* were harvested in July 2017 from Harbin, Heilongjiang Province, China. They were extracted by refluxing twice (2 h each time) with 70% ethanol. After filtration, the combined extract was concentrated under reduced pressure to provide the crude extract (CE). The crude extract was suspended in distilled water and partitioned successively with petroleum ether (60–90°C), ethyl acetate and *n*-butanol to afford after evaporation to dryness the petroleum ether, ethyl acetate, *n*-butanol and residual water extracts (corresponding to PEE, EAE, NBE, WE).

### 2.2 Animals and high-fat diet

The animal experiment was performed with Kunming mice (male, 16–20 g) purchased from Guangdong Medical Experimental Animal Center (Guangdong, China) (SCXK [Yue] 2013-0002). Mice were maintained in a specific-pathogen-free environment provided by Laboratory Animal Center of Gannan Medical University (SYXK [Gan] 2018-0004). They were reared in cages under controlled conditions (temperature 25 ± 2°C, relative humidity 60 ± 5%) with a 12-h light/dark cycle. All the mice had *ad libitum* access to diet and water during the entire experiment. High-fat diet (HFD) formula: sucrose 20%, lard 15%, cholesterol 1.2%, sodium cholate 0.2%, casein 10%, calcium hydrophosphate 0.6%, limestone ore powder 0.4%, premix 0.4%, basic feed 52.2%.

### 2.3 Experimental design

After 10 days of acclimation, mice were randomly divided into 18 groups, including blank control (BC), model (Mod), positive control (PC), and low-, medium-, and high-dose (corresponding to LD, MD, HD) groups of PEE, EAE, NBE, WE, and CE (corresponding to LDPEE, MDPEE, HDPEE; LDEAE, MDEAE, HDEAE; LDNBE, MDNBE, HDNBE; LDWE, MDWE, HDWE; LDCE, MDCE, HDCE), with 10 mice in each group. The BC group mice were fed with normal diet, while the other groups were fed with HFD. The PC group (simvastatin, 2.5 mg/kg body weight) and each extracts groups (200, 400, 800 mg/kg body weight corresponding to LD, MD, and HD groups) were administered by gavage. The BC group and Mod group were given equal volume of normal saline. Mice were gavaged once a day with 0.2 mL extracts or normal saline each time for four consecutive weeks. Body weight of mice was recorded once a week. After the last administration, all mice fasted for 12 h and drank freely. The next morning, after taking blood from the orbit, the mice were sacrificed by cervical dislocation, and then the liver, spleen and kidney were cut to prepare for the detection of relevant indicators. If there were significant difference (*p* < 0.05) between the four indexes of blood lipid (TC, TG, LDL-C, HDL-C) in the Mod group and the BC group, it indicates that the modeling was successful. All procedures with the animal experiment were approved by the ethics committee of Gannan Medical University.

### 2.4 Body weight and liver, spleen, kidney index in mice

The liver, spleen and kidney of mice were weighed and their indexes were calculated according to the following formula: liver index = liver weight/body weight × 100%; spleen index = spleen weight/body weight × 100%; kidney index = kidney weight/body weight × 100%.

### 2.5 Analysis of blood biochemical parameters

Blood obtained from mice orbit was centrifuged at 1,249 × g for 15 min, and the supernatant (serum) was taken for testing. The levels of TC, TG, LDL-C, HDL-C in serum were determined with a microplate reader (EPOCH, Bio Tek) and commercial kits (lot number: TC 20190114, TG 20190109, LDL-C 20190108, HDL-C 20190109; Nanjing Jiancheng Bioengineering Institute, Nanjing, China). The measurements were conducted in strict accordance with manufacturer’s instructions.

### 2.6 Liver function evaluation

#### 2.6.1 Detection index of liver injury

Measurements of ALT and AST in serum were made with commercially available kits (lot number: ALT 20190108, AST 20190108; Nanjing Jiancheng Bioengineering Institute, Nanjing, China) following the manufacturer’s instructions.

#### 2.6.2 Oxidative stress marker analysis in liver tissues

One hundred and fifty milligram of mice liver tissue was homogenized 3 times at 4°C with 1.35 mL of physiological saline (1:9, m/V), each time for 20 s. The liver tissue homogenate was centrifuged at 1,249 × g for 10 min and the supernatant was taken for the determination of liver indicators. The activity of SOD and the level of MDA were measured using commercial kits (lot number: SOD 20190114, MDA 20190112) purchased from Nanjing Jiancheng Bioengineering Institute (Nanjing, China), in strict conformity with the manufacturer’s instructions.

### 2.7 Histopathological analysis of liver

The liver difference in appearance, shape and color was compared by naked eye, and then histopathological examination was performed. A part of liver tissue was fixed in 4% paraformaldehyde solution, embedded in paraffin, sliced (4 μm thickness), stained with hematoxylin/eosin (H&E), and observed under an optical microscope (Eclipse Ci-L, Nikon, Tokyo, Japan).

### 2.8 Chemical composition analysis

#### 2.8.1 UPLC-Q/TOF-MS conditions

The UPLC-Q/TOF-MS were performed using a *Nexera X2* LC-30 AD UPLC system (Shimadzu Scientific Instruments, Marlborough, MA, United States), coupled to a SCIEX QTOF 6600 MS spectrometer (AB SCIEX, Framingham, MA, United States). The separation was performed on an ACQUITY UPLC HSS T3 C18 column (1.8 μm, 2.1 mm × 100 mm, Waters, Milford, MA, United States). The mobile phase was composed of water containing 0.1% formic acid (eluent A), acetonitrile containing 0.1% formic acid (eluent B). The gradient changes of mobile phases were 0–11 min, 5 ~ 90% B; 11–12 min, 90% B; 12–12.5 min, 90–5% B; 12.5–15 min, 5% B. The flow rate was 0.3 mL/min. The column temperature was set at 40°C and the injection volume was 5 μL.

The MS analysis was conducted using the time-of-flight (TOF) mass spectrometer (AB SCIEX, United States) with TurboIonSpray ion source, operating in the ESI positive and negative ionization full scan modes. The optimal parameters were as follows: Ion Source Gas1 (Gas1), 55; Ion Source Gas2 (Gas2), 55; Curtain gas (CUR), 35; Ion Source Temperature, 550°C; IonSapary Voltage Floating (ISVF), 5,500 V/−4,500 V; TOF MS scan m/z range, 50–1,500 Da; production scan m/z range, 25–1,000 Da; TOF MS scan accumulation time 0.25 s/spectra; product ion scan accumulation time 0.035 s/spectra. The secondary mass spectrometry was obtained using information dependent acquisition (IDA) and high sensitivity mode. Declustering potential (DP), ± 60 V (positive and negative ion mode); Collision Energy: 30 ± 15 eV.

SCIEX OS software was used to collect and process data. The software contains multiple confidence criteria, which including quality accuracy, retention time, isotopes and matching usage of compound library. Traditional Chinese Medicine (TCM) MS/MS Library could be searched and screened according to the first-order accurate mass number, isotope distribution ratio and MS/MS of compounds, and then the target compounds could be identified.

#### 2.8.2 GC-MS/MS conditions

The GC-MS/MS were carried out on 7890B-7000D gas chromatograph and triple quadrupole mass spectrometer (Agilent Technologies Inc., California, United States). The separation was performed on a DB-5MS quartz capillary chromatography column (30 m × 0.25 mm × 0.25 μm, Agilent, California, United States). The programmed temperature were as follows: the initial temperature was 60°C, maintained for 4 min, heated to 180°C at 5°C/min, maintained for 5 min, and then heated to 250°C at 3°C/min. The sample inlet temperature was 250°C. The carrier gas was helium and the flow rate was 1.2 mL/min. The pre column pressure was 72 kPa. The injection volume was 1 μL.

The MS conditions were as follows: the ion source was EI and the temperature was 230°C; the electron energy was 70 eV; the interface temperature was 270°C; the solvent delay time was 6 min, and the scanning quality range was 60–600 amu. The chemical components were identified by searching the NIST17.L mass spectrometry database and comparing it with standard spectra.

### 2.9 Statistical analysis

All animal experimental data were statistically analyzed with one-way analysis of variance (ANOVA) followed by Tukey’s HSD test using SPSS software (version 16, SPSS). The results were expressed as mean ± standard deviation (SD) (*n* = 10, calculated from 10 replicates) and considered as statistically significance when *p*-values were less than 0.05. The column plots were generated by GraphPad Prism (version 8.0, GraphPad Software).

## 3 Results

### 3.1 General behavioral observations and body weight changes in mice

During the experiment, the mice in each group showed good mental state and stable increased in body weight. There was no diarrhea and no animal death. There was no statistically significant difference in mice body weight between the treatment groups and the Mod group at 1, 2, 3, and 4 weeks of drug administration (Table 1).

**Table 1:** The effect of the aerial parts from *Allium sativum* on the body weight of mice.

Groups	Dose (mg/kg)	Body weight (g)			
		Week 1	Week 2	Week 3	Week 4
BC	–	40.19 ± 2.91	45.30 ± 2.93	48.44 ± 3.29	49.61 ± 3.67
Mod	–	41.51 ± 2.34	46.16 ± 2.92	48.58 ± 3.08	50.86 ± 3.77
PC	2.5	41.01 ± 3.45	46.39 ± 3.77	48.30 ± 3.95	51.08 ± 5.45
PEE	200	41.81 ± 2.13	46.45 ± 2.19	48.94 ± 2.27	51.15 ± 2.93
	400	40.01 ± 3.08	44.58 ± 4.04	46.79 ± 4.38	48.83 ± 5.01
	800	40.04 ± 4.83	44.00 ± 6.30	45.97 ± 7.22	47.10 ± 7.71
EAE	200	40.07 ± 2.84	44.93 ± 3.16	47.27 ± 3.22	50.53 ± 3.91
	400	41.64 ± 2.36	45.82 ± 3.21	47.01 ± 4.04	48.01 ± 4.51
	800	40.22 ± 2.15	44.71 ± 3.35	47.31 ± 3.48	50.03 ± 3.97
NBE	200	38.70 ± 3.04	42.79 ± 4.00	44.58 ± 4.17	47.89 ± 5.30
	400	39.85 ± 4.40	44.07 ± 5.45	46.13 ± 5.48	48.66 ± 6.21
	800	39.90 ± 3.24	44.17 ± 4.12	46.30 ± 4.77	49.35 ± 5.57
WE	200	39.73 ± 2.69	44.27 ± 3.13	45.99 ± 2.95	49.09 ± 3.01
	400	39.32 ± 3.34	43.99 ± 4.26	45.14 ± 4.22	47.38 ± 4.89
	800	41.60 ± 3.08	46.61 ± 3.96	48.06 ± 3.94	48.98 ± 4.11
CE	200	40.36 ± 2.90	45.52 ± 3.26	47.67 ± 4.16	49.94 ± 5.08
	400	38.81 ± 2.79	44.50 ± 3.61	46.43 ± 4.27	50.64 ± 4.93
	800	38.79 ± 3.20	43.02 ± 3.90	44.42 ± 4.37	45.80 ± 5.10

BC, blank control; Mod, model; PC, positive control; PEE, petroleum ether extract; EAE, ethyl acetate extract; NBE, n-butanol extract; WE, residual water extract; CE, crude extract. Values (body weight) are expressed as mean ± SD, *n* = 10.

### 3.2 Liver, spleen and kidney index in mice

The effects of the treatment groups on liver, spleen and kidney index in hyperlipidemic mice were shown in Table 2. Compared with the BC group, the liver index of the Mod group mice was remarkably increased (*p* < 0.01). Compared with the Mod group, the liver index in the high-dose groups of PEE, EAE, NBE, WE, and CE were remarkably reduced (*p* < 0.01), and the low-dose of PEE, NBE groups were significantly reduced (*p* < 0.05). There were no significant difference in spleen index and kidney index among each groups of mice.

**Table 2:** Liver index, spleen index and kidney index in mice.

Groups	Dose (mg.kg^−1^)	Liver index	Spleen index	Kidney index
BC	–	3.88 ± 0.25	0.207 ± 0.073	1.39 ± 0.10
Mod	–	5.00 ± 0.42^##^	0.236 ± 0.060	1.38 ± 0.17
PC	2.5	4.50 ± 0.33**	0.210 ± 0.079	1.25 ± 0.17
PEE	200	4.63 ± 0.22*	0.195 ± 0.065	1.27 ± 0.12
	400	4.59 ± 0.25*	0.191 ± 0.100	1.15 ± 0.09
	800	4.42 ± 0.17**	0.201 ± 0.066	1.37 ± 0.13
EAE	200	5.06 ± 0.50	0.244 ± 0.139	1.34 ± 0.18
	400	4.85 ± 0.25	0.276 ± 0.075	1.52 ± 0.28
	800	4.40 ± 0.75**	0.220 ± 0.042	1.24 ± 0.10
NBE	200	4.59 ± 0.22*	0.187 ± 0.069	1.27 ± 0.19
	400	4.58 ± 0.46*	0.213 ± 0.087	1.27 ± 0.19
	800	4.44 ± 0.73**	0.217 ± 0.088	1.21 ± 0.11
WE	200	5.25 ± 0.36	0.243 ± 0.052	1.38 ± 0.13
	400	4.65 ± 0.51	0.229 ± 0.082	1.28 ± 0.14
	800	4.37 ± 0.42**	0.212 ± 0.069	1.30 ± 0.18
CE	200	5.29 ± 0.34	0.230 ± 0.087	1.29 ± 0.17
	400	4.91 ± 0.76	0.262 ± 0.103	1.30 ± 0.17
	800	4.40 ± 0.25**	0.222 ± 0.068	1.31 ± 0.10

BC, blank control; Mod, model; PC, positive control; PEE, petroleum ether extract; EAE, ethyl acetate extract; NBE, n-butanol extract; WE, residual water extract; CE, crude extract. Data are expressed as mean ± SD, *n* = 10. ^##^*p* < 0.01, ^#^*p* < 0.05, compared with the BC group. ^*^p < 0.05, ^**^p < 0.01, compared with the Mod group.

### 3.3 The content of TC, TG, LDL-C, and HDL-C in serum

**Figure 1:**
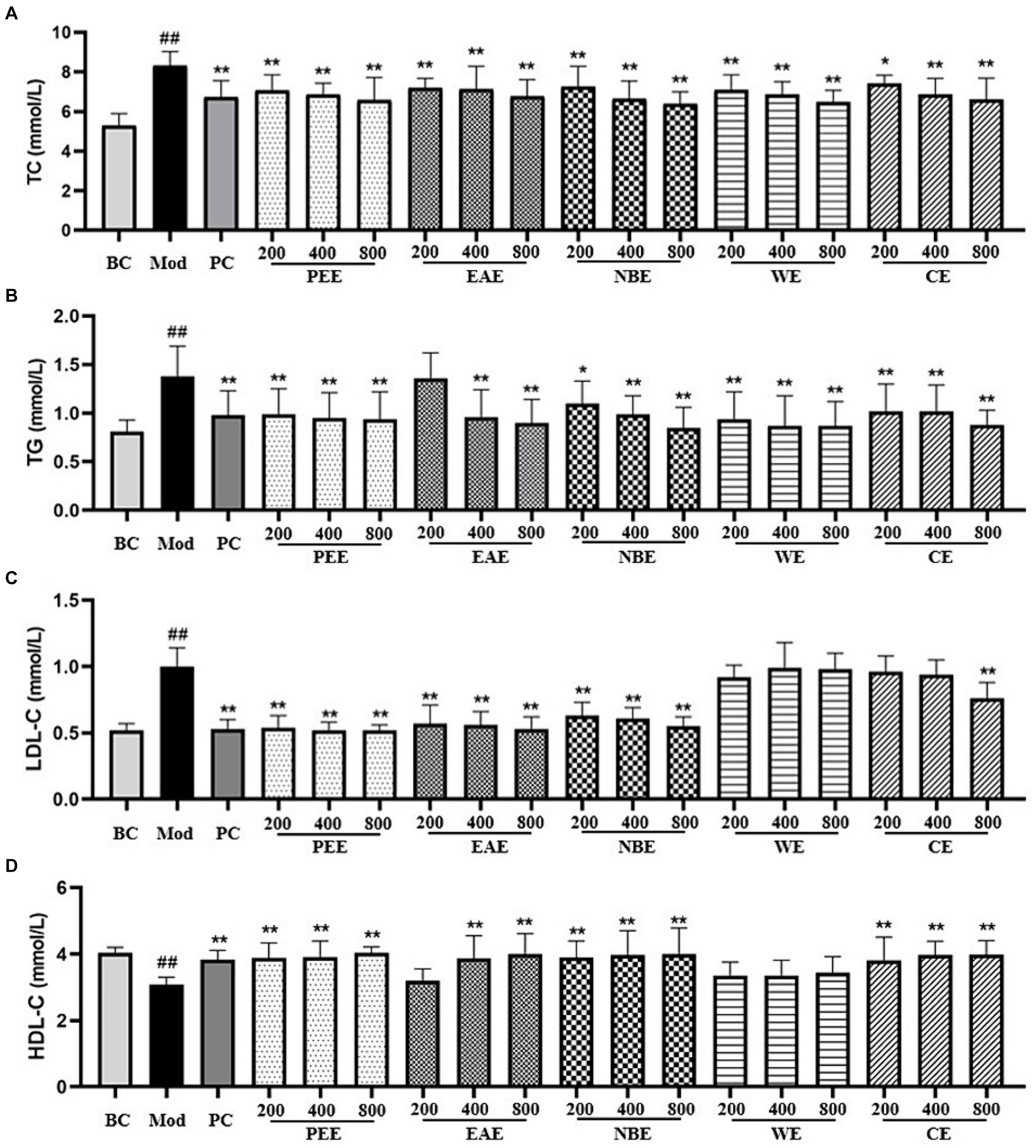
The effect of the aerial parts from *Allium sativum* on the content of serum TC, TG, LDL-C, and HDL-C in mice. BC, blank control; Mod, model; PC, positive control; PEE, petroleum ether extract; EAE, ethyl acetate extract; NBE, *n*-butanol extract; WE, residual water extract; CE, crude extract. **(A)** TC; **(B)** TG; **(C)** LDL-C; **(D)** HDL-C. Values are expressed as mean ± SD, *n* = 10; ^##^*p* < 0.01, ^#^*p* < 0.05, compared with the BC group; ^*^p < 0.05, ^**^p < 0.01, compared with the Mod group.

Compared with the BC group, the TC, TG and LDL-C content in the serum of the Mod group mice fed with HFD was remarkably increased (*p* < 0.01) (Figures 1A–C). Compared with the Mod group, except for the LDCE group (p < 0.05), the TC content in all other treatment groups were remarkably reduced (*p* < 0.01) (Figure 1A); there was no significant difference in TG content in the LDEAE group, while the other treatment groups showed extremely significant reductions (*p* < 0.01) (Figure 1B); the LDL-C content in the PEE, EAE, NBE, and HDCE groups were significantly reduced (*p* < 0.01), while there was no significant difference in the WE group (Figure 1C). In Figure 1D, compared with the BC group, the HDL-C content in the serum of the Mod group mice was significantly reduced (*p* < 0.01); compared with the Mod group, except for the LDEAE and WE groups (no significant difference), the HDL-C content in all other treatment groups was significantly increased (*p* < 0.01).

### 3.4 Liver function evaluation

**Figure 2:**
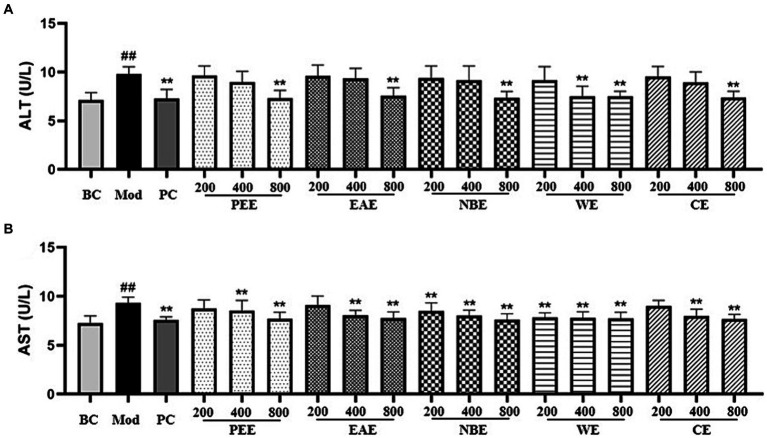
The effect of the aerial parts from *Allium sativum* on the content of serum ALT and AST in mice. BC, blank control; Mod, model; PC, positive control; PEE, petroleum ether extract; EAE, ethyl acetate extract; NBE, *n*-butanol extract; WE, residual water extract; CE, crude extract. **(A)** ALT; **(B)** AST. Values are expressed as mean ± SD, *n* = 10; ^##^*p* < 0.01, ^#^*p* < 0.05, compared with the BC group; ^*^p < 0.05, ^**^p < 0.01, compared with the Mod group.

ALT and AST are released after hepatocellular injury, making them useful markers for measuring liver function. Compared with the BC group, the AST activity in the serum of the Mod group mice was remarkably increased (*p* < 0.01); compared with the Mod group, the AST activity in the PC, HDPEE, HDEAE, HDNBE, MDWE, HDWE and HDCE groups were significantly reduced (*p* < 0.01) (Figure 2A). As shown in Figure 2B, the Mod group showed significantly increase (p < 0.01) in ALT activity, while the reverse trend were observed for PC, MDPEE, HDPEE, MDEAE, HDEAE, LDNBE, MDNBE, HDNBE, LDWE, MDWE, HDWE, MDCE, and HDCE groups (*p* < 0.01).

**Figure 3:**
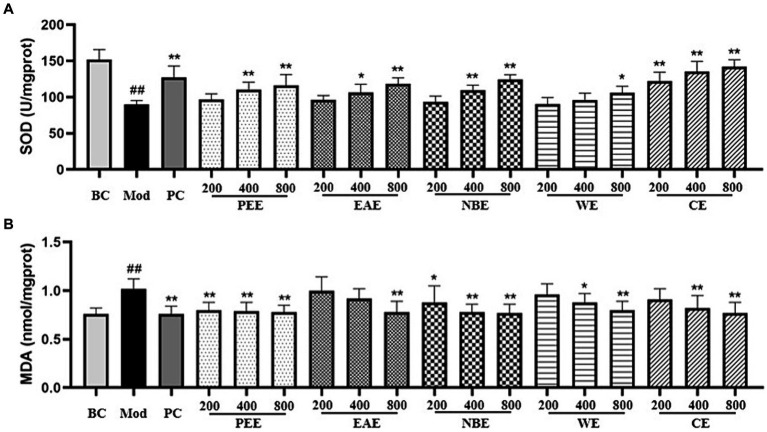
The effect of the aerial parts from *Allium sativum* on the activity of SOD and the level of MDA in mice. BC, blank control; Mod, model; PC, positive control; PEE, petroleum ether extract; EAE, ethyl acetate extract; NBE, *n*-butanol extract; WE, residual water extract; CE, crude extract. **(A)** SOD; **(B)** MDA. Values are expressed as mean ± SD, n = 10; ^##^*p* < 0.01, ^#^*p* < 0.05, compared with the BC group; ^*^p < 0.05, ^**^p < 0.01, compared with the Mod group.

The effects of the extracts on oxidative factors in hepatic tissues were shown in Figure 3. In comparison with the BC group mice, as shown in the figure, the activity of SOD in the liver tissue was reduced, and the level of MDA was increased of the Mod group mice (*p* < 0.01). Compared with the Mod group, the extracts could modulate the activity of SOD and the level of MDA. The SOD activity in the PC, MDPEE, HDPEE, MDEAE, HDEAE, MDNBE, HDNBE, HDWE and three doses of CE groups were significantly increased (*p* < 0.01 or 0.05) (Figure 3A); and except for the LDEAE, MDEAE, LDWE, and LDCE groups (no significant difference), the level of MDA in all other treatment groups was significantly reduced (*p* < 0.01 or 0.05).

### 3.5 Hepatic histopathological changes

**Figure 4:**
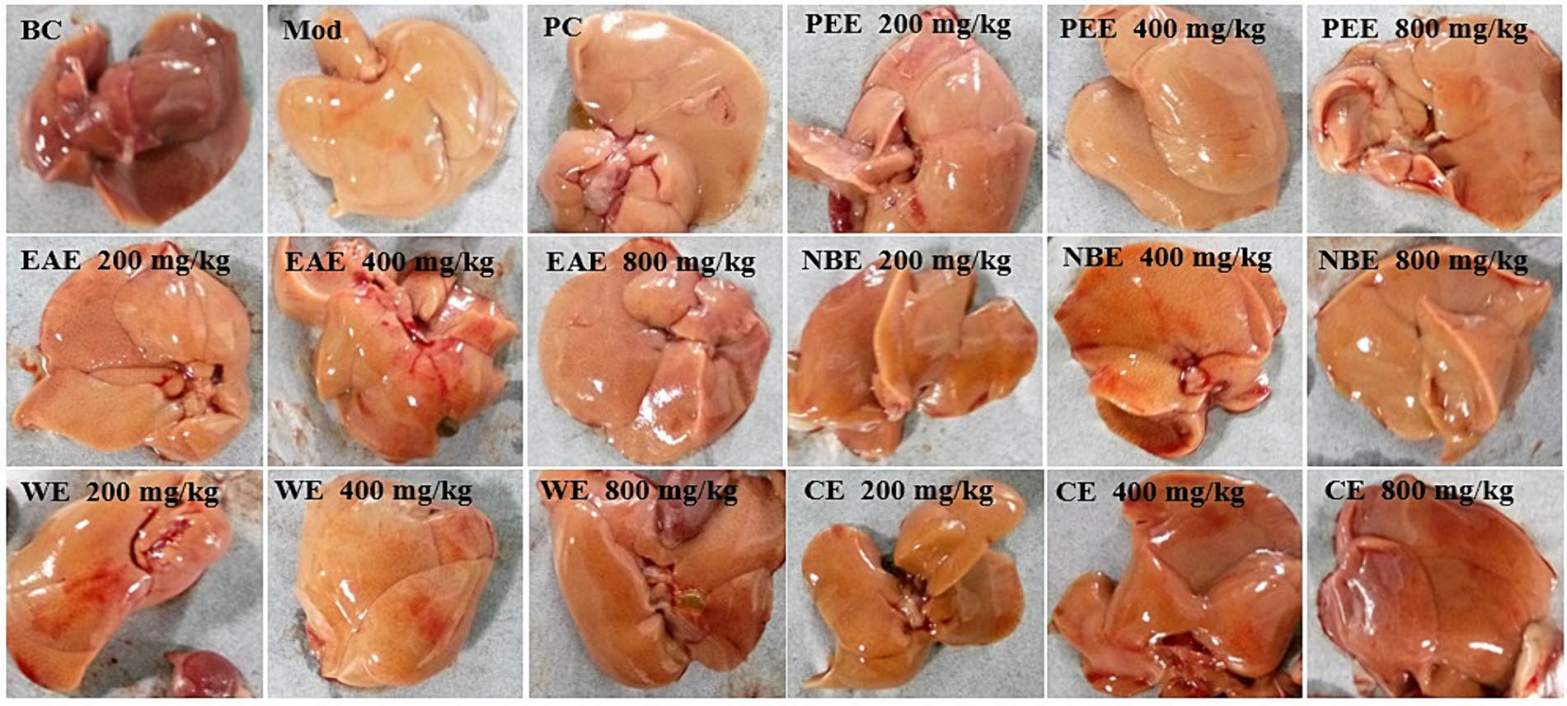
The effect of the aerial parts from *Allium sativum* on the appearance and morphology of the liver in hyperlipidemic mice. BC, blank control; Mod, model; PC, positive control; PEE, petroleum ether extract; EAE, ethyl acetate extract; NBE, *n*-butanol extract; WE, residual water extract; CE, crude extract.

After dissection, the liver of the mice was cut and observed with the naked eye. There were significant differences in the appearance and color of the liver between the BC group, Mod group, and administration groups. The liver of the BC group was reddish brown, smooth and delicate. The Mod group was yellow with visible fat particles. The morphology and color of each administration group showed varying degrees of improvement, as shown in Figure 4.

**Figure 5:**
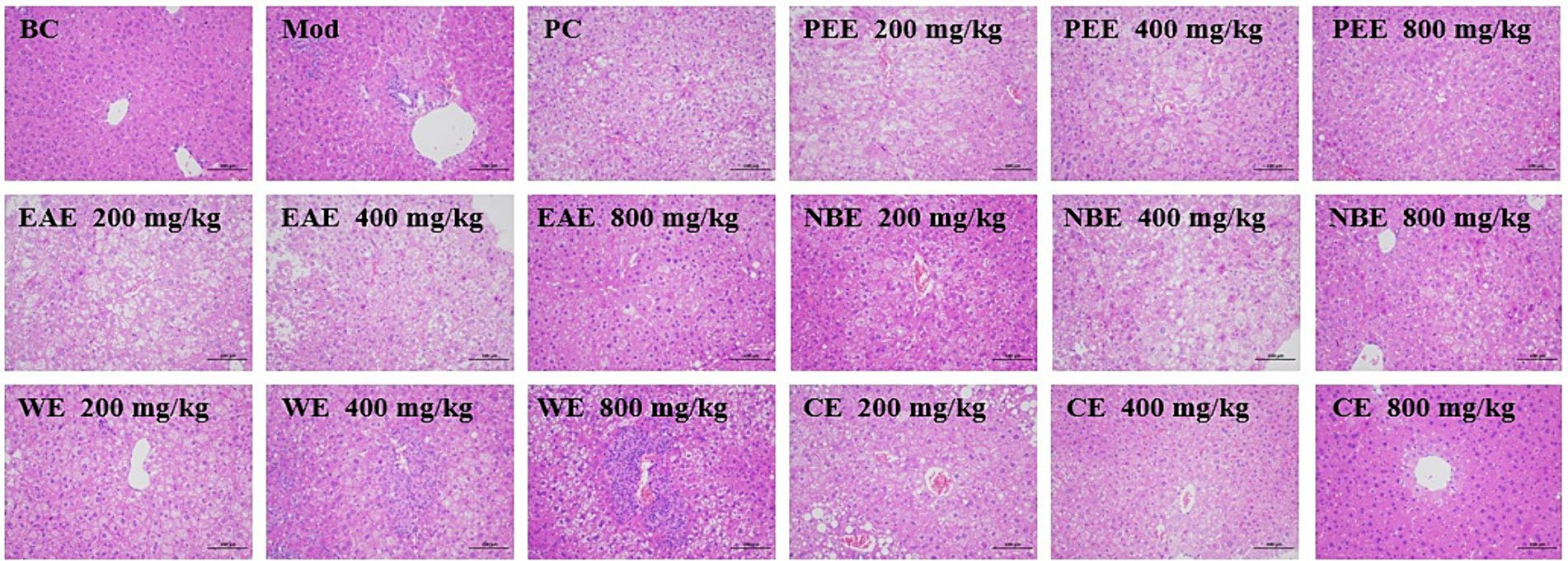
Histopathological examinations of liver tissues using H&E staining (200×). BC, blank control; Mod, model; PC, positive control; PEE, petroleum ether extract; EAE, ethyl acetate extract; NBE, *n*-butanol extract; WE, residual water extract; CE, crude extract. Values are expressed as the mean ± SD (*n* = 6) for all groups.

To ascertain the ameliorative efficacy of the aerial parts of *A. sativum* on the histopathological lesions in the liver tissues in mice with hyperlipidemia induced by HFD feeding, H&E staining experiment was performed. The histopathological examination results of the liver were shown in Figure 5. In the BC group, it was observed that the liver architecture of the mice was normal, without cellular steatosis, etc. While liver tissue injuries, including severe steatosis, vacuolization of hepatocytes, and heavy hepatocyte lipid droplet accumulation, were seen in the Mod group. The administration groups attenuated the pathological lesions in hepatic tissues; in particular, no significant lipid droplets were seen in the hepatocytes. So the aerial parts of *A. sativum* could improved liver tissue injuries of HFD-fed mice.

### 3.6 Chemical composition analysis

Using UPLC-Q-TOF/MS to analyze the NBE in the aerial parts of *A. sativum*, a total of 39 chemical components were identified, including 17 compounds in positive ion mode and 22 compounds in negative ion mode. As summarized in Table 3, 21 organic acids, including 2 aliphatic acids, 6 phenolic acids, and 13 other organic acids; 3 flavonoids; 15 nitrogen-containing components, including 3 alkaloids, 5 amino acids, 3 vitamins, and 4 nucleosides.

**Table 3:** The chemical constituents in *n*-butanol extract (NBE) of the aerial parts from *Allium sativum* identified by UPLC-Q/TOF-MS analysis.

No.	Name	RT (min)	Formula	Ion Mode	Exact Mass (*m*/*z*)	Library Score	Classification
1	Kaempferol	5.9	C_15_H_10_O_6_	[M-H]^−^	286.0477	100	A
2	L-Phenylalanine	1.9	C_9_H_11_NO_2_	[M + H]^+^	165.0790	100	B
3	Luteolin	4.0	C_15_H_10_O_6_	[M + H]^+^	286.0477	100	A
4	Linoleic acid	12.1	C_18_H_32_O_2_	[M-H]^−^	280.2402	100	C
5	Guanosine	1.5	C_10_H_13_N_5_O_5_	[M + H]^+^	283.0917	100	D
6	Alpha-Linolenic acid	11.4	C_18_H_30_O_2_	[M-H]^−^	278.2246	100	C
7	4-Hydroxybenzoic acid	2.9	C_7_H_6_O_3_	[M-H]^−^	138.0317	100	E
8	trans-Ferulic acid	4.0	C_10_H_10_O_4_	[M-H]^−^	194.0579	99.9	E
9	Undecanedioic acid	5.8	C_11_H_20_O_4_	[M-H]^−^	216.1362	99.8	F
10	L-Tyrosine	1.3	C_9_H_11_NO_3_	[M + H]^+^	181.0739	99.7	B
11	Azelaic acid	4.5	C_9_H_16_O_4_	[M-H]^−^	188.1049	99.7	F
12	Nicotinic acid	1.3	C_6_H_5_NO_2_	[M + H]^+^	123.0320	99.5	G
13	4-Pyridoxic acid	1.4	C_8_H_9_NO_4_	[M-H]^−^	183.0532	99.3	F
14	Caffeic acid	3.2	C_9_H_8_O_4_	[M-H]^−^	180.0423	99.3	E
15	Phthalic acid	3.1	C_8_H_6_O_4_	[M-H]^−^	166.0266	99.2	F
16	2-Isopropylmalic acid	2.8	C_7_H_12_O_5_	[M-H]^−^	176.0685	98.9	F
17	Adenosine	1.4	C_10_H_13_N_5_O_4_	[M + H]^+^	267.0967	98.8	D
18	Pyridoxine	1.3	C_8_H_11_NO_3_	[M + H]^+^	169.0739	98.8	G
19	5″-Methylthioadenosine	2.4	C_11_H_15_N_5_O_3_S	[M + H]^+^	297.0896	98.7	D
20	Isoferulic acid	4.0	C_10_H_10_O_4_	[M-H]^−^	194.0579	98.6	E
21	Tyramine	1.3	C_8_H_11_NO	[M + H]^+^	137.0841	98.4	E
22	Methylmalonic acid	1.2	C_4_H_6_O_4_	[M-H]^−^	118.0266	98.1	F
23	2-Methylglutaric acid	2.8	C_6_H_10_O_4_	[M-H]^−^	146.0579	98.0	F
24	Succinic acid	1.2	C_4_H_6_O_4_	[M-H]^−^	118.0266	97.5	F
25	Uridine	1.3	C_9_H_12_N_2_O_6_	[M + H]^+^	244.0695	97.3	D
26	2,2-Dimethylsuccinic acid	2.8	C_6_H_10_O_4_	[M-H]^−^	146.0579	96.8	F
27	Methylglutaric acid	2.8	C_6_H_10_O_4_	[M-H]^−^	146.0579	96.4	F
28	L-Leucine	1.3	C_6_H_13_NO_2_	[M + H]^+^	131.0946	96.1	B
29	Quercetin	5.3	C_15_H_10_O_7_	[M-H]^−^	302.0427	95.6	A
30	Thymine	2.0	C_5_H_6_N_2_O_2_	[M + H]^+^	126.0429	95.4	H
31	Phenyllactic acid	3.9	C_9_H_10_O_3_	[M-H]^−^	166.0630	94.9	F
32	6-Hydroxynicotinic acid	1.6	C_6_H_5_NO_3_	[M + H]^+^	139.0269	94.6	F
33	L-Norleucine	1.3	C_6_H_13_NO_2_	[M + H]^+^	131.0946	94.4	B
34	Salicylic acid	2.9	C_7_H_6_O_3_	[M-H]^−^	138.0317	94.0	E
35	N-Acetyl-L-phenylalanine	3.9	C_11_H_13_NO_3_	[M-H]^−^	207.0895	93.0	B
36	Sphinganine	7.9	C_18_H_39_NO_2_	[M + H]^+^	301.2981	92.9	H
37	L-3-Phenyllactic acid	3.9	C_9_H_10_O_3_	[M-H]^−^	166.0630	92.0	F
38	Riboflavin	3.2	C_17_H_20_N_4_O_6_	[M + H]^+^	376.1383	91.7	G
39	Indoleacrylic acid	2.5	C_11_H_9_NO_2_	[M + H]^+^	187.0633	90.5	H

A: Flavonoids; B: Amino acids and derivatives; C: Aliphatic acids; D: Nucleosides; E: Phenolic acids; F: other organic acids; G: Vitamins; H: Alkaloids.

In an attempt to identify the PEE in the aerial parts of *A. sativum*, it was proceeded with GC-MS/MS analysis. A total of 39 chemical components were identified, and the total peak area percentage was approximately 70% (with each compound having a peak area percentage greater than 0.10%). Among the identified chemical components, there were 1 organic sulfur compound, 26 aliphatic acids and derivatives, 2 other organic acids and derivatives, 3 aliphatic compounds, 2 alkaloids, and 5 phytosterols (Table 4).

**Table 4:** The chemical constituents in petroleum ether extract (PEE) of the aerial parts from *Allium sativum* identified by GC-MS/MS analysis.

No.	Name	RT (min)	Formula	Peak area percentage (%)	Classification
1	Diallyl disulfide	11.01	C_6_H_10_S_2_	0.16	A
2	9-Methoxy-3,4-dihydrophenanthren-1(2H)-one	26.78	C_15_H_14_O_2_	1.37	B
3	Chloroacetic acid, tetradecyl ester	27.18	C_16_H_31_ClO_2_	0.40	B
4	9-Methylene-1-phenyl-3,6-diazahomoadamantane	27.95	C_16_H_20_N_2_	0.40	C
5	Tetradecanoic acid	28.71	C_14_H_28_O_2_	0.15	B
6	Tetradecanoic acid, ethyl ester	29.52	C_16_H_32_O_2_	0.40	B
7	2-Pentadecanone, 6,10,14-trimethyl-	30.87	C_18_H_36_O	0.18	D
8	Ethyl 13-methyl-tetradecanoate	32.59	C_17_H_34_O_2_	0.10	B
9	Methyl palmitate	33.78	C_17_H_34_O_2_	0.17	B
10	Dibutyl phthalate	34.68	C_16_H_22_O_4_	3.31	E
11	n-Hexadecanoic acid	35.31	C_16_H_32_O_2_	4.58	B
12	Ethyl 9-hexadecenoate	35.46	C_18_H_34_O_2_	0.50	B
13	Hexadecanoic acid, ethyl ester	36.31	C_18_H_36_O_2_	4.86	B
14	2-Propenoic acid, 3-(3,4,5-trimethoxyphenyl)-, methyl ester	36.66	C_13_H_16_O_5_	0.11	B
15	(Z)-Ethyl heptadec-9-enoate	39.18	C_19_H_36_O_2_	0.26	B
16	Methyl 9-cis,11-trans-octadecadienoate	39.64	C_19_H_34_O_2_	0.15	B
17	Ethyl 15-methyl-hexadecanoate	39.79	C_19_H_38_O_2_	0.33	B
18	Phytol	40.23	C_20_H_40_O	0.49	D
19	9,12-Octadecadienoic acid (Z,Z)-	41.10	C_18_H_32_O_2_	3.18	B
20	9,12,15-Octadecatrienoic acid, (Z,Z,Z)-	41.24	C_18_H_30_O_2_	4.62	B
21	Dasycarpidan-1-methanol, acetate (ester)	41.39	C_20_H_26_N_2_O_2_	0.21	C
22	Linoleic acid ethyl ester	41.90	C_20_H_36_O_2_	5.87	B
23	9,12,15-Octadecatrienoic acid, ethyl ester, (Z,Z,Z)-	42.06	C_20_H_34_O_2_	6.32	B
24	Ethyl Oleate	42.34	C_20_H_38_O_2_	0.53	B
25	Octadecanoic acid, ethyl ester	43.05	C_20_H_40_O_2_	1.06	B
26	16-Hentriacontanone	48.19	C_31_H_62_O	12.41	D
27	9,19-Cyclolanost-24-en-3-ol, (3.beta.)-	49.85	C_30_H_50_O	5.73	F
28	Octan-2-yl palmitate	51.88	C_24_H_48_O_2_	0.43	B
29	Phthalic acid, di(oct-3-yl) ester	52.52	C_24_H_38_O_4_	0.59	E
30	.gamma.-Sitosterol	53.63	C_29_H_50_O	0.25	F
31	Docosanoic acid, ethyl ester	54.40	C_24_H_48_O_2_	0.66	B
32	24-Methylenecycloartan-3-one	55.70	C_31_H_50_O	0.81	F
33	9,12-Octadecadienoic acid (Z,Z)-, octyl ester	56.16	C_26_H_48_O_2_	0.23	B
34	9,12,15-Octadecatrienoic acid, 1-methylethyl ester, (Z,Z,Z)-	56.34	C_21_H_36_O_2_	0.18	B
35	Tetracosanoic acid, methyl ester	56.93	C_25_H_50_O_2_	0.28	B
36	9,19-Cyclolanostan-3-ol, 24-methylene-, (3.beta.)-	58.32	C_31_H_52_O	6.94	F
37	Phytyl heptadecanoate	59.00	C_37_H_72_O_2_	0.28	B
38	Pregn-4-ene-3,20-dione, 16,17-epoxy-,(16.alpha.)-	59.46	C_21_H_28_O_3_	0.10	F
39	Ethyl tetracosanoate	59.83	C_26_H_52_O_2_	0.43	B

A: Organic sulfur compounds; B: Aliphatic acids and derivatives; C: Alkaloids; D: Aliphatic Compounds; E: other Organic acids and derivatives; F: Phytosterols.

## 4 Discussion

Cardiovascular disease (CVD) is the most significant chronic disease worldwide that threatens human life and health. CVD (such as ischemic heart disease and ischemic stroke) dominated by atherosclerotic cardiovascular disease (ASCVD) is the first cause of death for urban and rural residents in China, accounting for more than 40% of the causes of death (38). In recent years, the disease burden of ASCVD in China has continued to increase (39), and the situation of prevention and control is severe. Epidemiological, genetic, and clinical intervention research evidence fully confirms that LDL-C is a pathogenic risk factor for ASCVD (40).

In the 20th century, the age standardized coronary heart disease mortality rate in the United States showed a turning point of decline since 1968, with a decrease of over 40% from 1980 to 2000. Among them, the contribution of controlling risk factors accounted for 44%, with the largest contribution being the decrease in TC levels, with a weight of 24% (41). However, data shows that the levels of TC, LDL-C, and TG among Chinese residents have significantly increased in 2012 compared to 2002, while HDL-C has significantly decreased. The incidence of dyslipidemia among people over 18 years old has significantly increased (38), while the awareness, treatment, and control rates of dyslipidemia among residents are at a relatively low level. Therefore, China is facing a continuous upward trend in the burden of ASCVD disease, and blood lipid management is urgent.

This article studies the preventive and therapeutic effects of the aerial parts of *A. sativum* on hyperlipidemia. Experiments have shown that the CE and their extracts (PEE, EAE, NBE, WE) of the aerial parts can reduce the levels of TC and TG in the serum of hyperlipidemic mice. The effects on the content of LDL-C and HDL-C are slightly different, with the WE being ineffective on both. The CE, PEE, EAE, and NBE can reduce the content of LDL-C to varying degrees and increase the content of HDL-C. In addition, the CE and their extracts can reduce the levels of ALT and AST in serum to varying degrees. The results of this study also showed that the CE and their extracts can increase SOD activity in the liver to varying degrees, and reduce MDA levels. The CE and their extracts can reduce the liver index of mice to varying degrees, but have no effect on the spleen index and kidney index.

Through comprehensive analysis of various indicators, the NBE and PEE of the aerial parts have the best preventive and therapeutic effects on hyperlipidemia, followed by the CE and EAE, and the WE has the worst effect. To explore the potential lipid-lowering chemical components in the aerial parts, the UPLC-Q/TOF-MS and GC-MS/MS were further used to analyze the chemical compositions of the NBE and PEE, respectively. A total of 78 compounds were identified, mainly including flavonoids, organic acids (such as aliphatic acids and phenolic acids), nitrogen-containing components (such as alkaloids, vitamins and amino acids) and phytosterols.

Literatures have reported that some ingredients in the NBE have lipid-lowering effects (Table 3). Firstly, studies have shown that flavonoids have lipid-lowering activity. Treatment of kaempferol (compound 1) (30 mg/kg and 150 mg/kg) for 6 and 10 weeks significantly lowered TC, TG, and LDL-C levels of rabbits in comparison to the model group (42). In addition, scholars have assessed the effect of quercetin (compound 29) on lipid profile induced by lindane in Wistar rats. Elevated levels of serum TC, TG, LDL, and tissue TC, TG with concomitant decrease in serum HDL and tissue phospholipids were decreased in lindane treated rats were found to be significantly decreased in the quercetin and lindane co-treated rats (43). The researchs showed that alpha-linolenic acid (compound 6) and linoleic acid (compound 4) could inhibit porcine adipogenesis by affecting preadipocyte proliferation, inhibiting differentiation and inducing apoptosis (44). Similar results were obtained in the study of primary cultures of human adipocytes and human preadipocyte cell line AML-I (45, 46). In addition to the above-mentioned flavonoids and organic acids having lipid-lowering effects, the nitrogen-containing components also have these effects. For example, riboflavin (compound 38) has a regulatory effect on lipid metabolism, which is manifested by inhibiting cholesterol biosynthesis, maintaining normal liver transport of lipids (47, 48), reducing levels of TC, LDL-C, and very low density lipoproteins (49), and preventing lipid peroxidation and accumulation (50–52). Therefore, riboflavin can play a role in the treatment of lipid metabolism and related diseases.

The PEE contains a large amount of aliphatic acids and derivatives, among which polyunsaturated fatty acids (PUFAs) account for a relatively high proportion, as shown in Table 4. In recent years, the incidence of hyperlipidemia has increased globally, and research on the lipid-lowering effects of PUFAs has attracted high attention from domestic and foreign scholars and made significant progress. PUFAs can regulate lipid metabolism by activating transcription factors and signaling pathways related to lipid metabolism (53–55), regulating inflammatory factors (56), increasing adiponectin levels (57), reducing leptin content and epigenetic modifications (58), thereby achieving the goal of lipid-lowering. The research findings provide reference for further development of drugs or functional foods derived from PUFAs, such as the aerial parts of *A. sativum*. In addition, the PEE also contains abundant plant sterols (phytosterols), such as 9,19-Cyclolanost-24-en-3-ol, (3.beta.)- (Cycloartenol) (compound 27), 9,19-Cyclolanostan-3-ol, 24-methylene-, (3.beta.)- (compound 36) and .gamma.-sitosterol (compound 30) (Table 4). Phytosterols and cholesterol have similar chemical structures and can inhibit intestinal cholesterol absorption (59, 60), thereby reducing plasma TC and LDL-C levels.

## 5 Conclusion

This article confirmed that the extracts of the aerial parts have good lipid-lowering effects, with the NBE and PEE having significant effects. Both the NBE and PEE can significantly reduce the content of TC, TG, LDL-C, ALT, and AST in mice serum, increase the content of HDL-C, and increase the activity of SOD in the liver, while reducing the level of MDA. Through chemical composition analysis, the results showed that the NBE is rich in organic acids, flavonoids, and nitrogen-containing components, while the PEE is mainly composed of aliphatic acids and derivatives, as well as plant sterols. The literature suggested that the above phytochemical components may be the active ingredients for lowering blood lipids. These investigations provided theoretical references for further development and utilization of the aerial parts of *A. sativum* resources.

## Data availability statement

The original contributions presented in the study are included in the article/supplementary material, further inquiries can be directed to the corresponding authors.

## Ethics statement

The animal study was approved by the Ethics Committee of Gannan Medical University. The study was conducted in accordance with the local legislation and institutional requirements.

## Author contributions

BH: Conceptualization, Funding acquisition, Investigation, Methodology, Project administration, Validation, Writing – original draft, Writing – review & editing, Data curation. HH: Data curation, Writing – review & editing. DP: Investigation, Resources, Writing – review & editing. ZW: Investigation, Resources, Writing – review & editing. QW: Funding acquisition, Project administration, Supervision, Writing – review & editing. HK: Conceptualization, Funding acquisition, Project administration, Supervision, Writing – review & editing.
